# Resveratrol Alleviates Endotoxin-Induced Myocardial Toxicity via the Nrf2 Transcription Factor

**DOI:** 10.1371/journal.pone.0069452

**Published:** 2013-07-22

**Authors:** Enkui Hao, Fangfang Lang, Yong Chen, Huilin Zhang, Xiao Cong, Xiaoqian Shen, Guohai Su

**Affiliations:** 1 Department of Cardiology, Jinan Central Hospital, Affiliated with Shandong University, Jinan, China; 2 Department of Obstetrics and Gynecology, Jinan Central Hospital, Affiliated with Shandong University, Jinan, China; 3 Department of Pharmacy, People’s Hospital of Rizhao, Rizhao, China; 4 Central Laboratory, Jinan Central Hospital, Affiliated with Shandong University, Jinan, China; University of Western Ontario, Canada

## Abstract

**Background/Aims:**

Septic cardiomyopathy is a severe condition that remains a challenge for clinical management. This study investigated whether the natural polyphenolic compound resveratrol could be used as a prophylactic treatment to alleviate sepsis-related myocardial injury; the underlying molecular mechanisms were deciphered by both *in vitro* and *in vivo* experiments.

**Methods:**

A mouse model of endotoxin-induced cardiomyopathy was developed by intraperitoneal injection of LPS, and resveratrol was administered prophylatically to the animals. Serum LDH and CK activities were measured to detect myocardial injury, and echocardiography was performed to monitor cardiac structure and function. Various cytokines/chemokines and the Nrf2 antioxidant defense system were examined in the heart tissue. The effects of resveratrol on LPS-induced Nrf2 activation, ROS generation, and apoptotic cell death were further investigated in cultured primary human cardiomyocytes. An Nrf2 specific siRNA was used to define its role in resveratrol-mediated cardiomyocyte protective effect.

**Results:**

Resveratrol pretreatment significantly attenuated LPS-induced myocardial injury in mice, which was associated with suppressed proinflammatory cytokine production and enhanced Nrf2 activation in the heart. In cultured primary human cardiomyocytes, resveratrol activated Nrf2, inhibited LPS-induced ROS generation, and effectively protected the cells from LPS-induced apoptotic cell death. Knockdown of Nrf2 abrogated resveratrol-mediated protection of the cells from LPS-induced cell death.

**Conclusion:**

Resveratrol effectively alleviates endotoxin-induced cardiac toxicity through mechanisms that involve the Nrf2 antioxidant defense pathway. Our data suggest that resveratrol might be developed as a useful prophylactic management for septic cardiomyopathy.

## Introduction

Septic shock is a syndrome of sepsis-induced organ failure and hypotension. Severe fatal hypotension and heart failure is one of the characteristics of septic shock. Depression of both left and right ventricular function and drop in left ventricle ejection fraction can occur during septic shock [1]. Cardiac depression in septic shock makes a complex clinical syndrome as it is difficult to maintain cardiac output. These resulted in reflex tachycardia and other compensatory physiological process to maintain systemic arterial blood pressure [2]. Patients with sepsis with lower left ventricular ejection fraction have increased plasma level of TNFα and apoptotic cell death of cardiomyocytes [3]. It has been demonstrated that addition of endotoxin lipopolysaccharide (LPS) from Gram negative bacteria in cardiomyocytes leads to apoptotic cell death [4]–[7]. Apoptotic cell death is also a contributing factor in other forms of heart failures such as myocarditis [8], congestive heart disease [9]–[12], diabetic cardiomyopathy [13], chronic pressure overload [14], [15] and ischemia-reperfusion injury [16], [17]. Actually, apoptosis represents a hallmark of cardiomyocyte injury due to endotoxin exposure during sepsis, and its critical role has been well recognized in the initiation and development of heart failure.

Resveratrol was identified in red wine as the source of anti-aging effect and lowing incidence of heart diseases as the result of researchers’ investigation of “French paradox”. Resveratrol is a polyphenolic compound that is present in many plant species and is especially abundant in food products such as grapes, peanuts, and mulberries. Interestingly, some Chinese herbs have been found to contain large amounts of resveratrol, and actually it is the major constituent of “Hu Zhang” (also known as Japanese Knotweed), which has been long used for its anti-aging property. Resveratrol has many pharmacological effects including beneficial effects in preventing heart diseases [18], [19]. Previously, resveratrol has been shown to have protective effect in apoptotic cell death in cardiomyocytes [20]–[22]. SIRT1, the founding member of sirtuin family, was found to be involved in the protective effect of resveratrol in cardioprotection [23]. The antioxidant properties of resveratrol are also important for its cardioprotective effects [24]. In vascular smooth muscle cells, resveratrol has been shown to induce activation of nuclear erythroid-related factor 2 (Nrf2) [25]. Nrf2 is a member of the cap-N-collar family, which is the principal transcription factor that regulates antioxidant response element-mediated expression of detoxifying antioxidant enzymes [26], [27].

In this study we investigated whether resveratrol has any cardioprotective effects in endotoxin-induced myocardial injury. By utilizing a mouse model, we demonstrated that resveratrol treatment significantly attenuated LPS-induced myocardial injury in mice, which was associated with suppressed proinflammatory cytokine production and enhanced Nrf2 activation in the heart. In cultured primary human cardiomyocytes, resveratrol activated Nrf2, inhibited LPS-induced ROS generation, and effectively protected the cells from LPS-induced apoptotic cell death. Knockdown of Nrf2 abrogated resveratrol-mediated protection of the cells from LPS-induced cell death, suggesting an important role of Nrf2 in the cardioprotective effects of resveratrol.

## Materials and Methods

### Mice and Treatments

Male C57BL/6 mice of 6–8 weeks old were obtained from the Experimental Animal Center of Shandong University (Jinan, Shandong, China). Resveratrol and LPS were purchased from Sigma in China (Beijing, China). Resveratrol was dissolved in DMSO:Tween 80:normal saline (10∶10:180), and LPS was dissolved in normal saline. Both reagents were administered intraperitoneally (i.p.) at a volume of 10 ul/gram mouse.

All animal care and experimental protocols were in compliance with the Animal Management Rules of the Health Ministry of the People’s Republic of China (document No 55, 2001). The study was approved by the Institutional Animal Care and Use Committee (IACUC) of Shandong University.

### Serum Creatine Kinase (CK) and Lactate Dehydrogenase (LDH) Measurements

Serum CK and LDH levels were determined using an automated analyzer (Abbott Architect, Abbot Park, Illinois, USA).

### Echocardiography

Mice were lightly anesthetized with isoflurane mixed with oxygen. Echocardiographic measurements were performed by using the VisualSonics Vevo770 system (VisualSonics, Inc, Toronto, Canada), which is equipped with a 30- MHz mechanical scan that can obtain high resolution 2-dimensional images. B mode images were obtained in the plane containing aortic and mitral valves. M mode images were obtained from the parasternal short-axis view at the level of papillary muscles. LV end-diastolic diameters and ejection fraction were calculated by using Vevo Analysis software.

### Cell Culture and Treatment

Human neonatal cardiomyocyte (HCM) were purchased (Shanghai FuMeng Gene Bio-technology Co., LTD) and were cultured in cardiomyocyte medium provided. HCM were maintained in tissue culture incubator at 37°C under 5% CO2 atmosphere. The cells were treated by LPS for the indicated concentrations for 24 h. Cells were pretreated with resveratrol for 3 h prior to LPS treatment.

### Cell Viability Assay

Cell viability was analyzed by MTT assay. For this assay, equal numbers of cardiomyocytes were plated on 96-well plates and maintained in regular growth medium for 2 days. The cells were then subjected to LPS and resveratrol treatment when indicated, as described earlier. MTT reagents (final concentration, 0.625 mg/mL) were added to each well and incubated at 37°C for 4 h, and the cells were lysed with acidic isopropanol (0.04N HCl). After incubation at room temperature for 15 minutes, the plates were then analyzed with a multi well ELISA reader at 570 and 650 nm.

### Reverse Transcription and Real-time PCR

In the present study, the expression of NRF-2 (nuclear factor, erythroid derived 2), HO-1 (heme oxygenase 1), GCLM (glutamate-cysteine ligase ), TNFa (tumor necrosis factor), IL1β (interleukin 1 beta), MIP1α (macrophage inflammatory protein-1alpha), MCP1(CD46) or β actin were detected by reverse transcription (RT) and real-time PCR. Total RNA was extracted from tissue or cells using Trizol total RNA isolation reagent (Invitrogen) as detailed by the instructions from manufactures. For detection of expression of the genes, the quantitative polymerase chain reaction primers were purchased from SABiosciences. PCR condition was 5 min at 95°C of denature temp, 40 cycles of 30 s at 95°C, 30 s at 60°C of annealing temp, and 30 s at 72°C, and ended with 10 min at 72°C of extension temp. The fold changes were calculated based on relative quantification method.

### Cell Transfection with NRF-2 siRNA

Nrf2 siRNA and scramble control were purchased from Shanghai GenePharma (Shanghai Zhangjiang Hi-Tech Park, Shanghai, China). The cultured cardiomyocytes were transfected with 50 nM of the siRNA for 24 hours using the supplied transfection reagents according to manufacturer’s instructions. The cells were then changed to fresh medium and were treated with resveratrol followed by LPS treatment.

### Immunoblotting Analyses

Nuclear extraction for Nrf2 and HO-1 was performed as follows. The tissue was homogenized in ice-cold lysis buffer (10 mmol/L HEPES, pH 7.9, 10 mmol/L KCl, 0.1 mmol/L EDTA, 1 mmol/L DTT, 0.1 mmol/L EGTA) for 15 min. After adding NP-40, centrifuged 10,000 rpm at 4°C for 3 min, the supernatant was collected as cytoplasmic protein. The pellets were homogenized in ice-cold lysis buffer (20 mmol/L HEPES, pH 7.9, 400 mmol/L NaCl, 1 mmol/L EDTA, 0.1 mmol/L EGTA) for 15 min, and was then centrifuged 12,000 rpm at 4°C for 10 min. The supernatant was collected as the nuclear protein and added PMSF to the final concentration of 1 mmol/L. The protein concentration of nuclear extract was determined using a BCA Protein Assay reagent kit (Novagen, Madison, WI, USA). Nuclear extract aliquots of 50 µg was separated by SDS/PAGE, transferred 2 h on to PVDF membranes, and the non-specific binding of antibodies was blocked with 5% non-fat dried milk in PBS. Membranes were then probed with polyclonal rabbit anti-Nrf2 antibody (1∶200, Santa Cruz Biotechnology) and Histone H3 (1∶200, Santa Cruz Biotechnology) overnight at 4°C. After four washes with TPBS, HRP-conjugated goat anti-rabbit second antibody (1∶2000, Rockland, Gilbertsville) was incubated with membranes for 1 h at room temperature. The relative density of bands was analysed on a X-ray film exposure. The densitometric values were normalized with respect to the values of actin immunoreactivity to correct for any loading and transfer differences between samples.

### Immunocytochemistry for Detection of Nrf2 Localization

Immunocytochemistry was performed for Nrf2 localization in response to LPS and resveratrol in cardiomyocyte. Human cardiomyocyte were seeded at 1×10^4^ cells/well in glass chamber slides and cultured overnight at 37°C. Cells were then treated as described for 24hours. At the end of incubation, cells were washed twice in PBS and fixed in 4% paraformaldehyde for 10 min at room temperature. Cells were permeabilized with 0.1% Triton X-100, washed, and blocked with 10% BSA for 1 h. Cells were then incubated overnight with rabbit polyclonal anti-Nrf2 antibody (1∶200 dilution in 1% BSA in PBS) in a humidified chamber at 4°C. Cells were washed with PBS and incubated with FITC-labeled anti-rabbit IgG diluted 1∶200 in 1% BSA for 1 h at room temperature in the dark, and were counterstained with DAPI dye to show the nuclear morphology. After the slides were rinsed with PBS, coverslips were applied and the cells were photographed using a fluorescence microscope.

### Flow Cytometry Detection of Cell Death

Cardiomyocytes were digested with trypsin solution for 10 min at 37°C. Cells were collected and washed twice with cold PBS. 5 µl of Annexin V (Pharmingen, San Diego, CA, USA) and 5 µl of Sytox Green (Life technologies) were added to the cells, which were resuspended in 500 µl 1× binding buffer. The cells were gently vortexed and incubated for 15 min at room temperature in the dark, then they were analyzed by flow cytometry within 1 h. Annexin V labeled with a fluorophore could identify cells in the early stage of apoptosis, and Sytox Green, a fluorescent nucleic acid binding dye, was responsible for staining cells in the medium and late stages of apoptosis. Analysis was based on gating a subpopulation of cells by forward scatter versus side scatter.

### Caspase 3 Acitivity Assay

Caspase 3 acitivity was measured from tissue extracts by a Caspase-3/CPP32 Colorimetric Assay Kit (Biovision). The assay is based on spectrophotometric detection of the chromophore p-nitroaniline (pNA) after cleavage from the labeled substrate DEVD-pNA. The pNA light emission was quantified using plate reader at 405-nm. The fold increase in CPP32 activity was determined by comparison of the absorbance of pNA from an apoptotic sample with an uninduced control.

### Statistical Analysis

Data were expressed as mean ± standard error of the mean, and statistical analysis was done by using SPSS 16.0.2 software (Chicago, IL, USA). One-way analysis of variance followed by Tukey’s Post Test for multiple group comparisons. P<0.05 was considered statistically significant.

## Results

### Resveratrol Attenuates Endotoxin-induced Myocardial Injury in Mice

**Figure 1:**
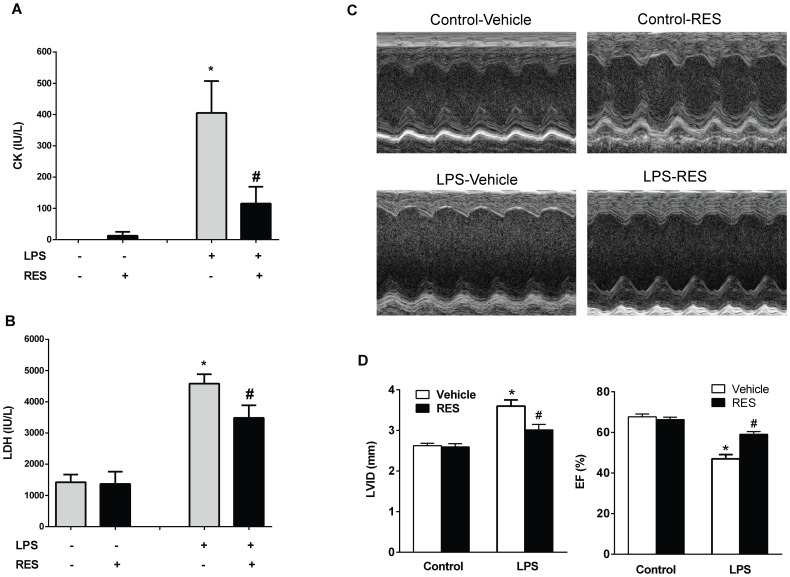
Resveratrol attenuates LPS induced myocardial injury in mice. C57BL/6 mice were injected intraperitoneally (i.p.) with 10 mg/kg of resveratrol or a vehicle once a day for totally 3 days, followed by i.p. injection of 4 mg/kg of LPS. The mice were sacrificed 18 hrs after LPS challenge. ***A*** and ***B***, CK and LDH levels in the serum samples were measured. (n = 6 mice/group, *P<0.05 versus vehicle treated control group, #P<0.05 versus LPS treated group). Cardiac function was assessed by echocardiography. ***C*** *,* Representative M-mode echocardiograms. ***D***, End-diastolic left ventricular inner dimension (LVID) and LV ejection fraction (EF) were calculated. n = 5 per group, *P<0.05 versus vehicle treated control group, #P<0.05 versus LPS treated group.

To examine whether resveratrol was able to confer cardiac protective effects in vivo, we treated C57BL/6 mice intraperitoneally with 10 mg/kg of resveratrol, a dose commonly used for animal experiments (28, 29). The mice were then challenged with 4 mg/kg of LPS overnight for about 18 hours, which caused marked myocardial tissue damage as evidenced by elevated serum creatine kinase (CK) and lactate dehydrogenase (LDH). Resveratrol treatment significantly reduced LPS-induced elevation of CK and LDH in the mice (Figure 1A, B). Left ventricular (LV) structure and function were assessed by echocardiography. As shown in Figure 1C and 1D, LPS caused an increase in end-diastolic left ventricular inner dimension (LVID) and a decrease in ejection fraction (EF), which was significantly attenuated by pre-treatment with resveratrol. The heart rates of the mice among all the four treatment groups were not statistically different (Table 1).

**Table 1:** Heart rates of mice subjected to echocardiography.

Animal groups	Control+Veh	Control+Res	LPS+Veh	LPS+Res
**Heart rate (bpm)** *	489.3±7.77	486.7±11.75	457.5±9.11	469.2±7.04

* bpm, beats per minute. There is no statistical difference of the heart rates among all the treatment groups.

### Resveratrol Suppresses Endotoxin-induced Production of Pro-inflammatory Cytokines and Activation of Nrf2 Antioxidant Defense Pathway in vivo

**Figure 2:**
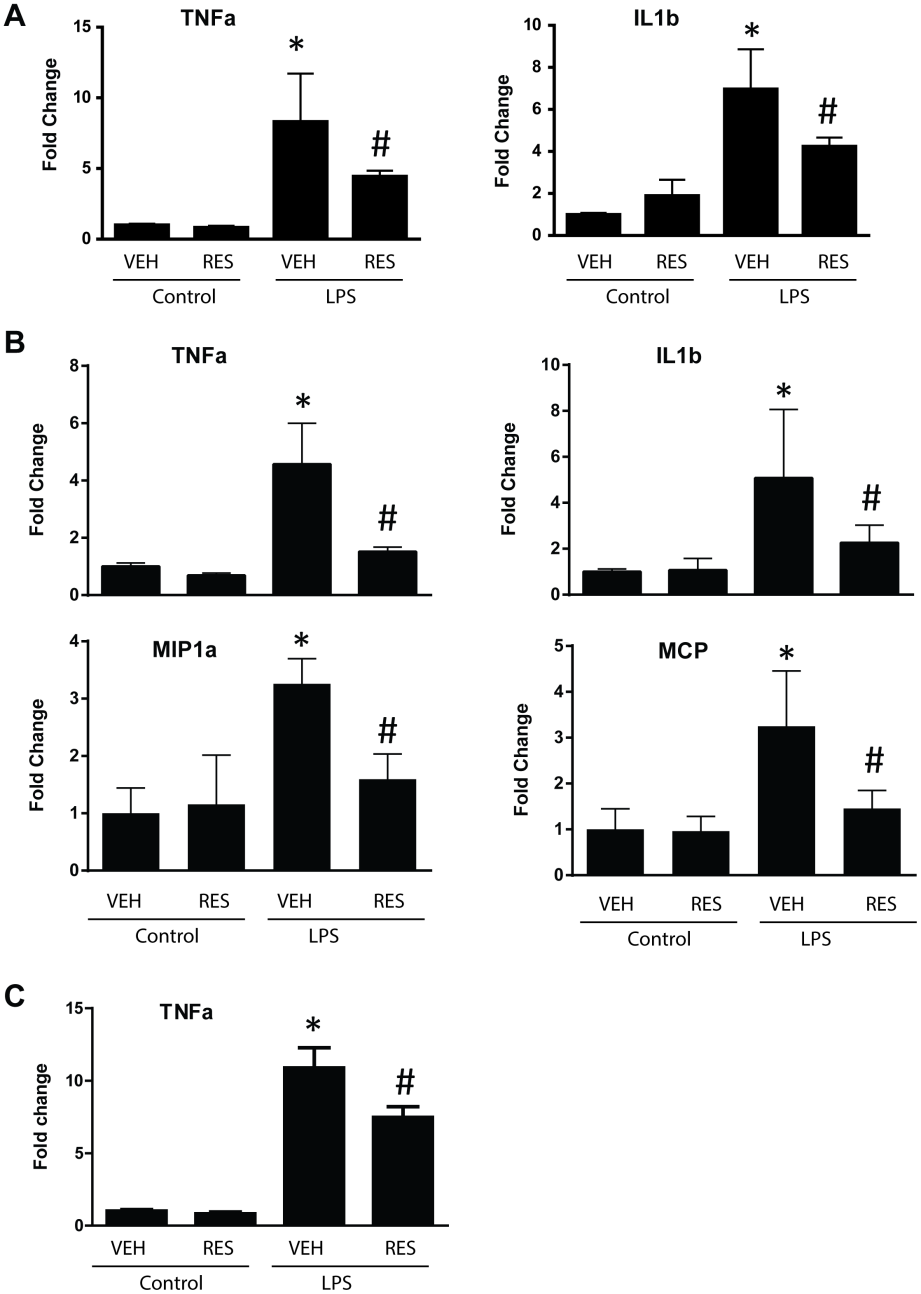
Resveratrol attenuates LPS induced production of pro-inflammatory cytokines and chemokines. C57BL/6 mice were injected i.p. with 10 mg/kg of resveratrol or a vehicle once a day for totally 3 days, followed by i.p. injection of 4 mg/kg of LPS. The mice were sacrificed 4 h (***A***) and 18 h (***B***) after LPS challenge, and the heart tissues were collected to prepare RNA samples. Real time RT-PCR was performed to detect expression of TNF-α, IL-1β, MIP-1α, and MCP. (n = 6 mice/group, *P<0.05 versus vehicle treated control group, #P<0.05 versus LPS treated group). ***C***, Human primary cardiomyocytes were pre-treated with 3 µM of resveratrol for 3 h, followed by incubation with 3 µg/ml of LPS for 4 h. TNF-a mRNA was quantified by real-time RT-PCR. (n = 4/group, *P<0.05 versus vehicle treated control group, #P<0.05 versus LPS treated group).

**Figure 3:**
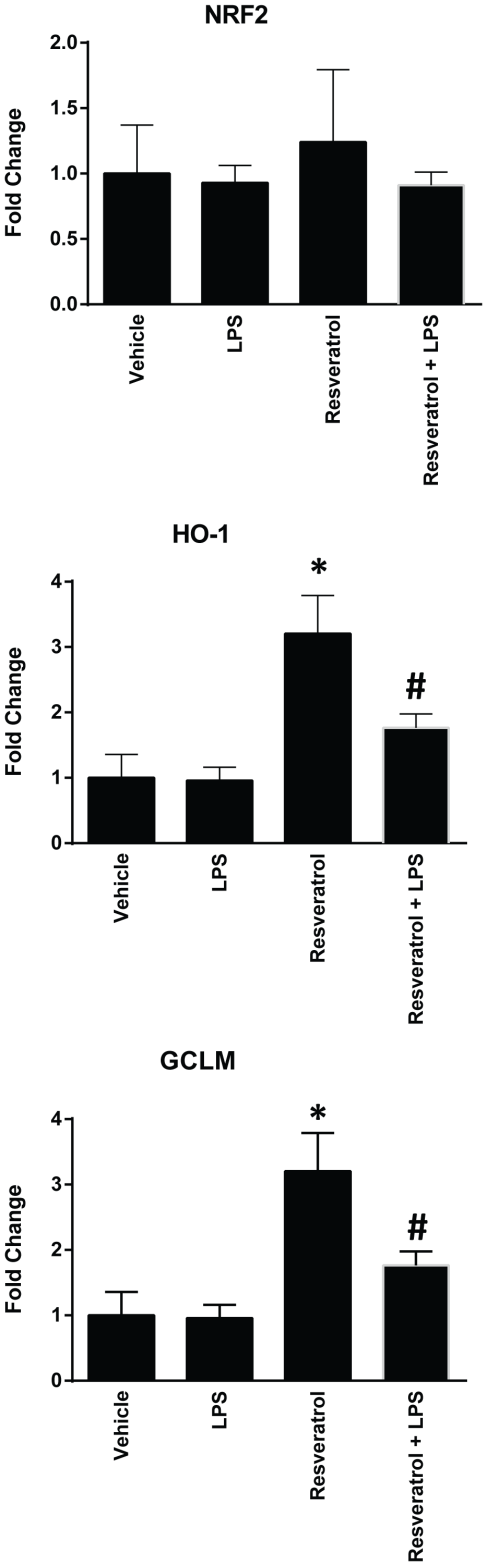
Resveratrol pretreatment inhibits LPS induced Nrf2 activation in vivo. C57BL/6 mice were injected i.p. with 10 mg/kg of resveratrol or a vehicle once a day for totally 3 days, followed by i.p. injection of 4 mg/kg of LPS. The mice were sacrificed 18 hrs after LPS challenge, and the heart tissues were collected to prepare RNA samples. Real time RT-PCR was performed to detect expression of Nrf2 and its target genes HO-1 and GCLM. (n = 6 mice/group, *P<0.05 versus vehicle treated control group, #P<0.05 versus LPS treated group).

Consistent with its myocardial protection, resveratrol treatment significantly inhibited endotoxin-induced production of pro-inflammatory cytokine TNF-α and IL-1β, and chemokine MIP-1α and MCP (Figure 2A, 2B). LPS treatment also directly induced TNF-α production in cultured cardiomyocytes, which was suppressed by resveratrol pretreatment (Figure 2C). In the heart tissues, resveratrol administration was able to induce Nrf2 activation evidenced by the increased expression of its target gene HO-1 and GCLM as shown in Figure 3.

### Resveratrol Directly Protects against LPS-induced Apoptotic Cell Death of Cardiomyocytes

**Figure 4:**
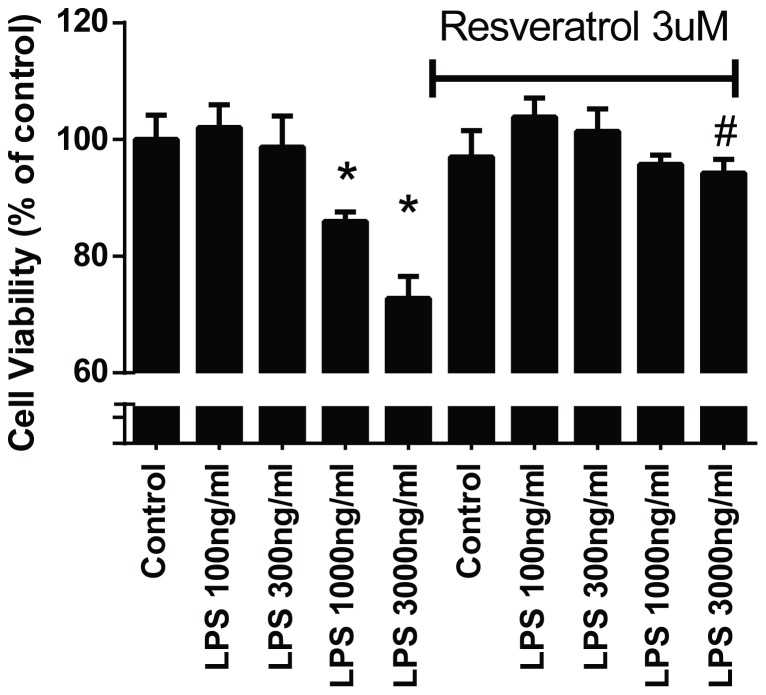
Resveratrol protects against LPS-induced cell death of cultured human primary cardiomyocytes. The cells were cultured for 2 days, and were then treated with 3 µM of resveratrol for 4 hrs, followed by incubation for 24 h with various concentrations of LPS. Cell viability was analyzed by MTT cell viability assay. *p<0.05 vs Control, #p<0.05 vs the corresponding LPS treatment, n = 4/group.

**Figure 5:**
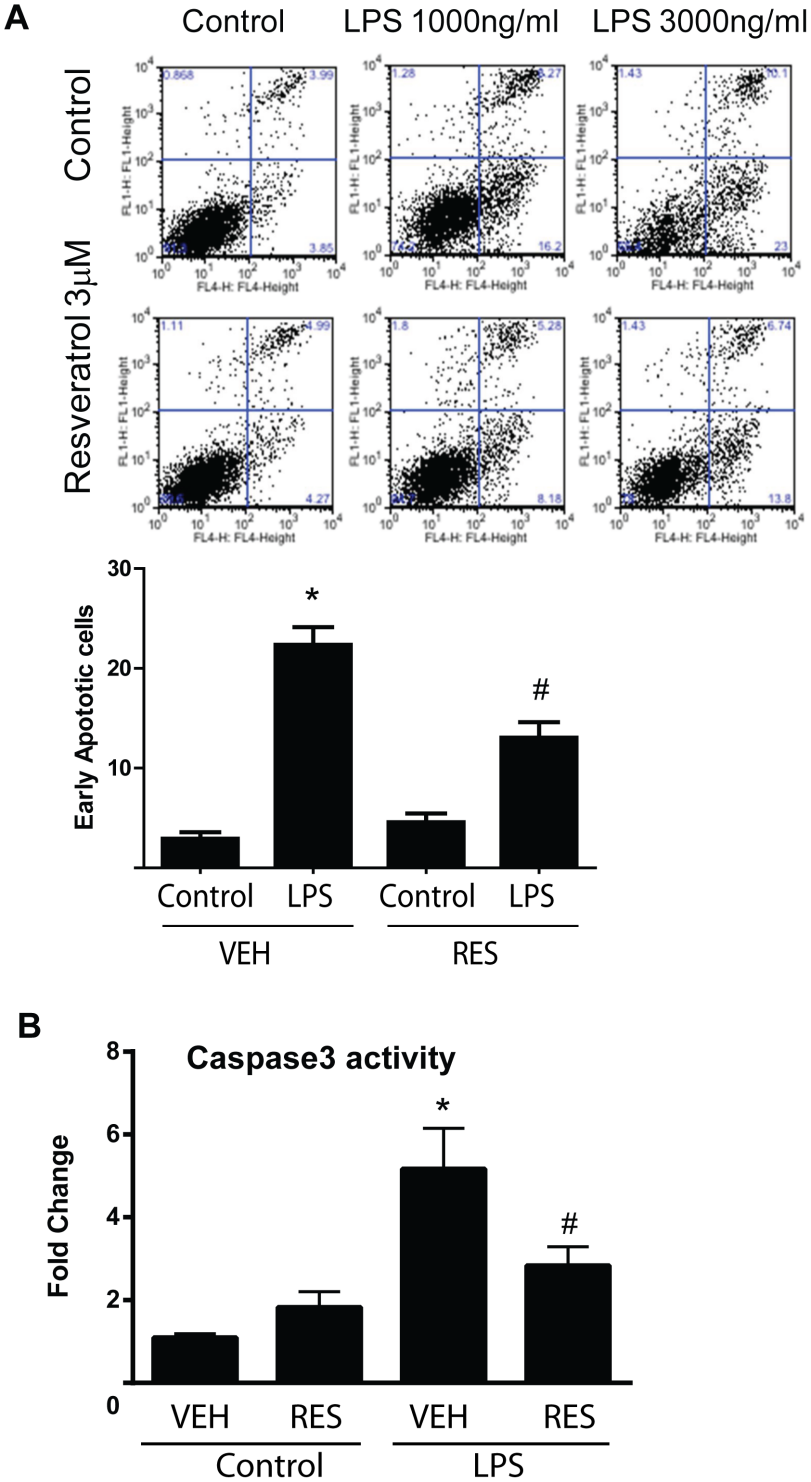
Resveratrol attenuated LPS induced apoptotic cell death of cultured human primary cardiomyocytes. *****A*****, LPS-induced apoptotic cell death in cardiomyocytes was determined by flow cytometry with Annexin V/Sytox green assay. Representative scatter plots of Sytox Green (y axis) vs Annexin V (x axis) were shown. The lower right quadrants represent the annexin V-positive and Sytox Green-negative apoptotic cells. Quantification data were shown in the lower panel (n = 4/group, *p<0.05 vs Control, #p<0.05 vs LPS treatment only). ***B***, C57BL/6 mice were injected i.p. with 10 mg/kg of resveratrol or a vehicle once a day for totally 3 days, followed by i.p. injection of 4 mg/kg of LPS. The mice were sacrificed 18 h after LPS challenge. The heart tissues were collected and the tissue extracts were used to assay caspase 3 activity by using a Caspase-3/CPP32 Colorimetric Assay Kit. (n = 6 mice/group, *P<0.05 versus vehicle treated control group, #P<0.05 versus LPS treated group).

To investigate whether cardiomyocytes could be directly influenced by resveratrol, we cultured human primary cardiomyocytes and treated them with LPS for 24 hours with or without resveratrol pretreatment. As shown in Figure 4, LPS treatment at 1000 and 3000 ng/ml caused significant cell death, which was reversed by pretreatment with resveratrol. We have performed a pilot dose dependent study for resveratrol and found that resveratrol at 3 µM offered optimal cell death protection in our culture system (data not shown). We further performed flow cytometry analysis with the early apoptotic marker annexin V, and found that the number of apoptotic and necrotic cells was significantly lower in resveratrol pretreated cells compared with the cells treated with LPS only (**Figure 5A**). To determine whether resveratrol could reduce LPS-induced apoptosis in the heart, we performed caspase 3 activity in the heart tissue extracts. As shown in Figure **5B**, resveratrol pretreatment significantly inhibited LPS-induced capase 3 activation in the heart.

### Resveratrol Alleviates Mitochondrial Reactive Oxygen Species (ROS) Production Induced by LPS in Cardiomyocytes

**Figure 6:**
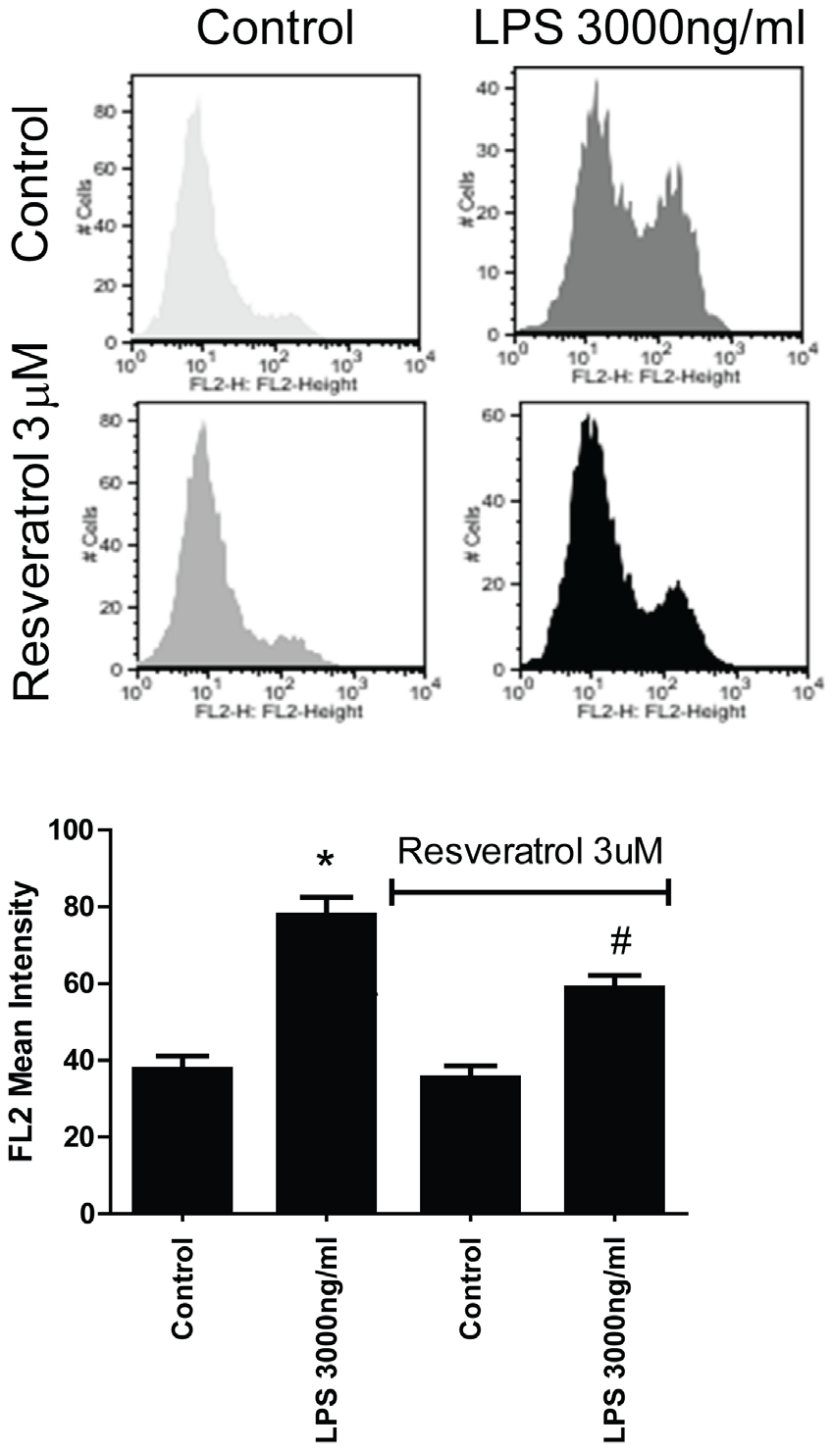
Resveratrol reduces LPS-induced mitochondrial ROS production in cardiomyocytes. **Mitochondrial-specific ROS assays were performed by flow cytometry as described in Methods.** Quantification of mean intensity was presented as a bar graph shown in the lower panel. *p<0.05 vs Control, #p<0.05 vs LPS treatment, n = 4/group.

To test that resveratrol might be able to attenuate oxidative stress in LPS-treated cardiomyoctes, we conducted flow cytometry using MitoSOX Red staining to measure mitochondrial ROS production, which is an important source of cellular ROS and represents one of the key events in apoptotic cell death. We found that LPS at 3000 ng/ml induced mitochondrial ROS production up to 2–fold, which was attenuated significantly by resveratrol treatment (**Figure 6**).

### Nrf2 Plays an Important Role in Resveratrol-mediated Protection against Endotoxin-induced Cardiomyocyte Toxicity

**Figure 7:**
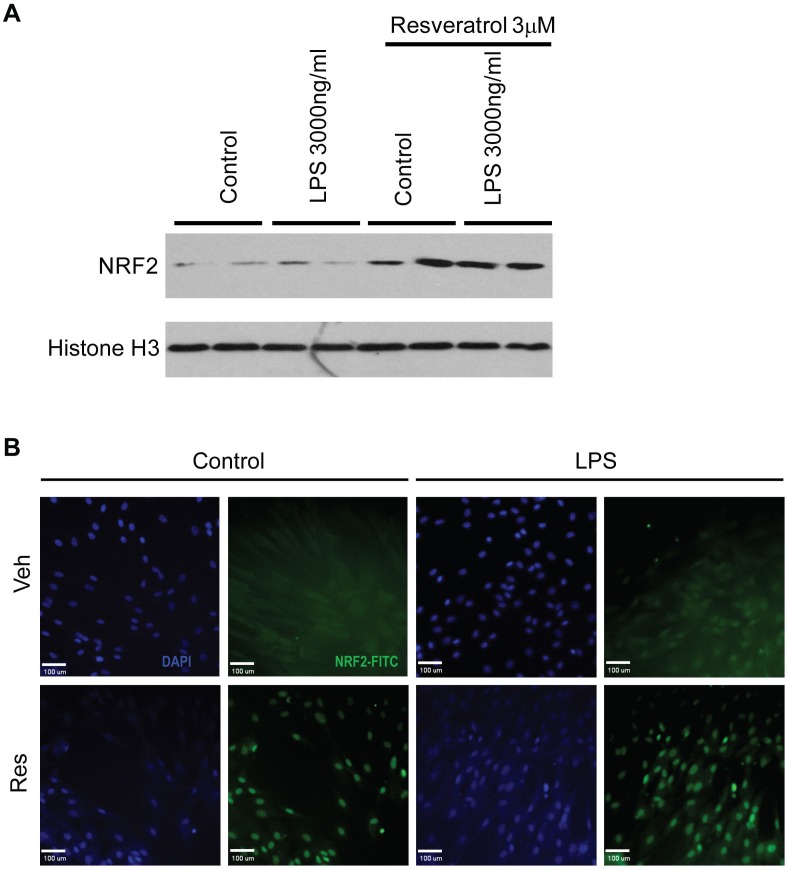
Resveratrol upregulated NRF2 protein nuclear accumulation in cardiomyocytes. *****A*****, Nrf2 protein expression in cell nuclei of cardiomyocytes was measured by Western blot analysis in the Control, LPS, Resveratrol, and Resveratrol+LPS treated groups. The representative results of Western blots of Nrf2 are shown. The nuclear protein histone H3 was detected to confirm the nuclear preparation and equal protein loading. ***B***, Immunocytochemistry was performed for Nrf2 localization in response to LPS and resveratrol in cardiomyocyte. Nrf2 protein was visualized with an FITC-labeled antibody, and the nuclear morphology was visualized with DAPI dye. (Original magnification 100×).

**Figure 8:**
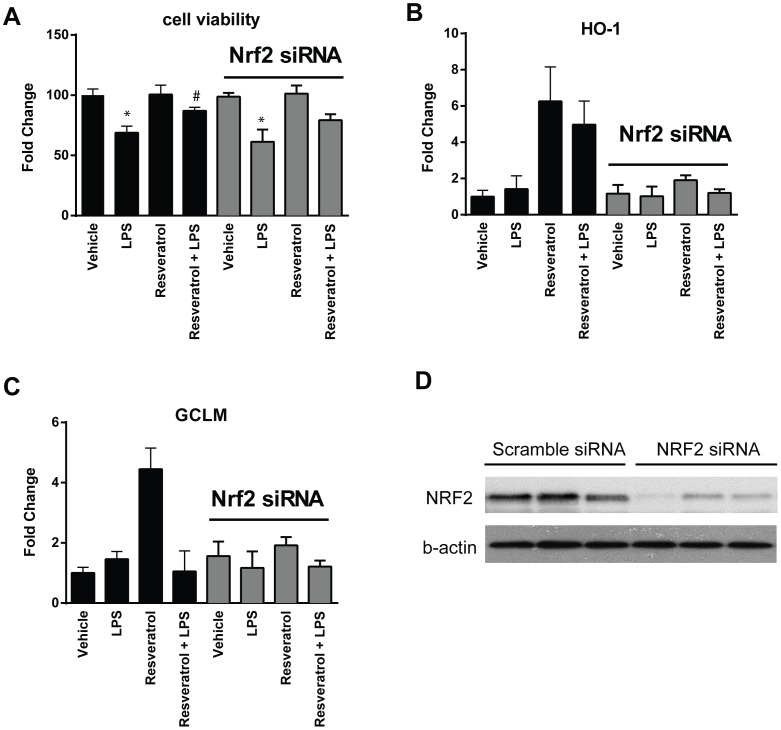
Knockdown of Nrf2 abrogates resveratrol-mediated protection of LPS induced cell death in human cardiomyocytes. The cells were transfected with a scramble control siRNA or Nrf2 siRNA, and were then treated with resveratrol, followed by LPS. Cell viability was analyzed by MTT assay (***A***). *p<0.05 vs Control, #p<0.05 vs the corresponding LPS treatment, n = 4/group. The cell expression of Nrf2 target gene HO-1 and GCLM were measured by real-time RT-PCR (***B and C***). Knockdown of Nrf2 protein in the cells treated with resveratrol was confirmed with Western Blot analysis of the nuclear extracts (***D***).

As shown in Figure 3, resveratrol treatment in vivo led to activation of Nrf2, which is an important antioxidant defense mediator. We performed further experiments to determine whether Nrf2 was responsible for resveratrol-mediated protection in LPS-induced cell death. Western blotting analysis using nuclear protein fractions revealed higher levels of Nrf2 protein nuclear accumulation in cardiomyocytes treated with resveratrol, which was confirmed by immunofluorescence staining of the cultured cells (**Figure 7**). We then transfected the cells with an siRNA specifically targeting Nrf2, which completely inhibited Nrf2 expression and activation in the cells as evidenced by the inhibited expression of HO-1 and GCLM (**Figure 8**). And importantly, knockdown of Nrf2 abrogated resveratrol-mediated protection of the cells from LPS-induced cell death (**Figure 8**).

## Discussion

As an important organ system frequently affected by sepsis and always affected by septic shock, the cardiovascular system and its dysfunction during sepsis have been studied in clinical research for more than six decades. First report demonstrated in 1951 where the research group recognized a hyperdynamic state with full bounding pulses, flushing, fever, oliguria, and hypotension [30]. Sepsis patients with myocardial depression are at a 50–70% greater risk of mortality than patients without cardiovascular complications [31]. There are a number of available preventive measures such as prophylactic antibiotics, maintenance of normoglycemia, selective digestive tract decontamination, vaccines, and intravenous immunoglobulin. Once sepsis is manifested, prompt and adequate antibiotic therapy accompanied by surgical removal of the infectious focus, if indicated and feasible, is the mainstay and also the only strictly causal line of therapy [32]. Despite all these modalities, however, it remains a big clinical challenge for effectively manage the disease to minimize mortality. Numerous basic research and clinical trials have been undertaken to curb the lethal toll of sepsis through modulation of this uncontrolled immune response [33], [34]. Research data have accumulated that suggest that inhibitors of 3-hydroxy-3-methylglutaryl coenzyme A reductase, or statins, have therapeutic benefits independent of cholesterol lowering, termed “pleiotropic” effects [35]. However the key mechanistic event in cardiovascular complication in sepsis is apoptosis [36]. The process of apoptosis involves multiple cell types including cardiomyocytes and endothelial cells and the molecular pathways of apoptosis in those cells are still controversial [37]–[45].

LPS, a complex glycoprotein that resides in the outer membranes of Gram-negative bacteria, has been implicated as a causative agent in apoptosis in cardiomyocytes and endothelial cells, a pivotal event that can lead to septic shock and associated syndromes. LPS is a potent stimulator of pro-inflammatory cytokine production, particularly TNFα [46]. It has also been reported that the release of LPS into the circulation induces endothelial apoptosis in vivo and thus causes microvascular injury in numerous tissues, including the lung, gut, and liver, during sepsis [47]. Tissues and organs obtained from patients who have died of sepsis and multi-organ failure and animal models of endotoxemia and sepsis reveal enhanced apoptotic cell death [47]. Here we have demonstrated that LPS induced apoptotic cell death in cardiomyocytes, consistent with earlier reports of cardiomyocyte death from other organisms [6], [37], [39], [48]. We showed that pretreatment with resveratrol leads to protection against LPS-induced myocardial damage in mice and apoptotic cell death in cardiomyocytes. We also showed that LPS stimulation results in mitochondrial ROS production in human cardiomyocytes and the production of mitocondrial ROS is significantly reduced by resveratrol pretreatment. These data are in agreement with reports that LPS induces TNFα production in cardiomyocytes, which in turn produces ROS in several other model systems [37], [38], [49], [50]. And interestingly, there is evidence also supporting that LPS-induced mitochondrial ROS production in cardiomyocytes can in turn play a role in TNF-alpha expression in the cells (51). Mitochondria are known as one of the key sources of such ROS production, and it has been shown that resveratrol can reduce mitochondrial ROS production in other types of cells [52].

Resveratrol has been shown to be beneficial in many diseases such as cancer, heart diseases and metabolic syndrome [53]. It is known that the nuclear factor Nrf2 is bound to Kelch-like ECH-associated protein-1 (Keap1) in the cytoplasm under normal conditions. Under oxidative stress or other potentially damaging stimuli, Nrf2 is released from Keap1 and translocates to the nucleus, where it binds to antioxidant response element (ARE) sequences, leading to the transcriptional activation of anti-apoptotic genes [54]. We tested in this study whether Nrf2 was involved in the cardiac protection by resveratrol for endotoxin-induced myocardial injury. We found that Nrf2 increased significantly in the heart tissue and the cultured cardiomyocytes upon resveratrol treatment. Similarly to our finding, a recent study showed that the traditional Chinese medicine Si-Wu-Tang can also activate Nrf2 [55]. Since Nrf2 is a very important endogenous antioxidant, it is possible that the increased Nrf2 activity might mediate resveratrol-induced attenuation of the mitochondrial ROS production in the cardiomyocytes. Resveratrol was recently shown to activate SIRT1, a NAD(+)-dependent deacetylase, that leads to an improved mitochondrial function, which in turn activates the transcription factor Nrf2 that coordinates expression of key antioxidant mechanisms by binding to the antioxidant response elements [23], [25], [56]–[58]. Sirt1 has been previously shown to be an important endogenous inhibitor of ROS production and apoptosis in cardiomyocytes (59, 60). Resveratrol was also known to block TNF-α induced inflammation in fibroblast through SIRT1 (61). Therefore, similarly to these previous reports, resveratrol-induced Nrf2 expression observed in our study might be also mediated by Sirt1. Importantly, we showed in our study that knockdown of Nrf2 expression in the cardiomyocytes made resveratrol no longer able to attenuate LPS-induced cellular toxicity, suggesting that Nrf2 indeed plays a critical role in the cardioprotection of resveratrol.

In summary, we found that prophylactic treatment with resveratrol significantly alleviated endotoxin-induced myocardial injury in mice, which was associated with suppressed proinflammatory cytokine production and enhanced Nrf2 activation in the heart. In cultured primary human cardiomyocytes, resveratrol activated Nrf2, inhibited LPS-induced ROS generation, and effectively protected the cells from LPS-induced apoptotic cell death. Knockdown of Nrf2 abrogated resveratrol-mediated protection of the cells from LPS-induced cell death, suggesting an essential role of Nrf2 in the cardioprotection of resveratrol. Our findings suggest that resveratrol might be developed as a useful prophylactic management for septic cardiomyopathy.
